# Breathing technique‐dependent acute cardiopulmonary responses during squats in healthy females

**DOI:** 10.14814/phy2.70831

**Published:** 2026-03-19

**Authors:** Sonja Hummelmann, Maxi Kramer, Ulrich Laufs, Sven Fikenzer, Johannes Lässing, Roberto Falz

**Affiliations:** ^1^ Institute of Sports Medicine and Prevention University of Leipzig Leipzig Germany; ^2^ Clinic for Cardiology, University of Leipzig Medical Center Leipzig Germany; ^3^ Department of Exercise Science and Sports Medicine Martin Luther University Halle‐Wittenberg Halle (Saale) Germany; ^4^ Human‐Machine‐Interaction Magdeburg‐Stendal University of Applied Science Magdeburg Germany

**Keywords:** blood pressure response, breath‐holding, cardiovascular response, controlled breathing, heart rate, squat, strength training

## Abstract

Breath‐holding (BH), including variations of the Valsalva maneuver, is used during resistance exercise to stabilize the trunk and increase intra‐abdominal pressure. However, its hemodynamic and ventilatory effects during low‐to‐moderate intensity strength training in women remain unclear. In a randomized crossover design, 17 healthy, young females performed squats at three intensities (bodyweight, 25%, and 50% of 10‐repetition maximum) under two breathing conditions: BH or controlled breathing (CB) during the concentric phase. Cardiopulmonary responses were assessed using impedance cardiography, spirometry, and auscultatory arterial blood pressure measurement. Across intensities, BH elicited significantly higher heart rate (+2%–5%; *p* < 0.01), cardiac output (+3%–8%; *p* = 0.02), systolic blood pressure (+16%–23%; *p* < 0.01), rate‐pressure product (+20%–30%; *p* < 0.01), and rating of perceived exertion (+14%–19%; *p* < 0.01) than CB. Oxygen uptake (−11%–15%; *p* < 0.01) and minute ventilation (−16%–17%; *p* < 0.01) were significantly reduced during BH, followed by a post‐exercise overshoot. Stroke volume and blood lactate did not differ significantly. Even at low‐to‐moderate intensities, BH during resistance exercise induces moderate cardiovascular strain and transient ventilation reductions. In healthy, young women, these responses remained within a non‐critical range, suggesting that short, controlled BH may represent a viable and safe strategy during strength training.

## 1 INTRODUCTION

Strength training is an important recommendation for physical activity in healthy individuals (Chaabene et al., 2025; Garber et al., 2011; Westcott, 2012; World Health Organization, 2018) and has proven benefits in the exercise therapy of diverse internal diseases (Alizaei Yousefabadi et al., 2021; Li et al., 2024; Strasser et al., 2013; Virani et al., 2023; Whelton et al., 2018). Small‐weight or bodyweight exercises are commonly used in strength endurance training, especially in the recreational and fitness sectors, as well as for prevention and rehabilitation (Bennie, De Cocker, et al., 2020; Bennie, Kolbe‐Alexander, et al., 2020).

Breath‐holding (BH) is a natural reflex during high‐effort resistance exercises. In strength training, a forced expiratory motion (abdominal press) with inhibited exhalation (closed glottis) can occur consciously or unconsciously (MacDougall et al., 1992; McCartney, 1999). The application of BH also occurs in recreational and rehabilitation sports, particularly during full‐body exercises that engage the core muscles even without additional load (Finnoff et al., 2003). Literature often equates this type of exhalation under exertion with the Valsalva maneuver (VM) (Hackett & Chow, 2013), which is defined by a voluntary forced expiratory effort against a closed airway (Pstras et al., 2016). By increasing intra‐abdominal and intrathoracic pressure, the maneuver enhances core stability and relieves lumbar spine load during lifting tasks (Blazek et al., 2019; Daggfeldt & Thorstensson, 1997; Hemborg et al., 1985). However, the VM transiently reduces venous return and stroke volume (SV) while simultaneously elevating peripheral resistance and sympathetic activity, increasing heart rate (HR) and arterial blood pressure (Goldstein & Cheshire, 2017; Looga, 2005). Minute ventilation (V̇_E_) and oxygen uptake (V̇O_2_) are transiently reduced, followed by a compensatory post‐maneuver overshoot. Overall, the physiology and pathophysiology of single VM applications beyond physical training are well documented and influenced by factors such as maneuver duration and technique, exercise intensity and repetitions, age, gender, body position, and training experience (Drury & Green, 2023; Olschewski & Brück, 1990; Pstras et al., 2016). Mean arterial blood pressure recovery takes almost 10 s after one VM (Delaney et al., 2020). However, the cardiovascular regulations associated with a single VM are not fully applicable to repeated and intermittent BH during physical activity. Due to its association with alterations in blood pressure and HR, VM or BH is not recommended for patients with cardiovascular risk factors (Blazek et al., 2019; Hackett & Chow, 2013; Niewiadomski et al., 2012; Pstras et al., 2016).

In strength training, cardiovascular regulation is also affected by repetitive muscle contractions. Blood pressure fluctuates with each lift but increases progressively with each repetition. Nevertheless, the use of BH or VM resulted in a significantly greater increase in blood pressure than slow exhalation during the execution of high‐load resistance tasks (MacDougall et al., 1992; Narloch & Brandstater, 1995). Submaximal and maximal leg press exercises (85% of the 5‐repetition maximum (RM) to 100% of 1‐RM) with VM could cause a HR peak up to 186 bpm, a (intra‐arterial) systolic blood pressure (SBP) up to 307–345 mmHg, and a diastolic blood pressure of 238–284 mmHg (Haykowsky et al., 2001; MacDougall et al., 1985; Narloch & Brandstater, 1995; Palatini et al., 1989). These hemodynamic responses are more pronounced at higher exercise intensities and higher VM pressures (Chigira et al., 2024; Drury & Green, 2023; MacDougall et al., 1985; Narloch & Brandstater, 1995). However, even at low intensities, BH or VM during resistance exercise of male test subjects resulted in a significantly higher HR, blood pressure, and rate‐pressure product than other breathing techniques (Chigira et al., 2024; Finnoff et al., 2003; Hughes et al., 1989; Linsenbardt et al., 1992). BH during high‐intensity strength training enhances core stability (Hackett & Chow, 2013), but it also leads to a marked increase in blood pressure (Haykowsky et al., 2001). In contrast, Lepley and Hatzel (2010) found no significant differences in HR and blood pressure when performing bench press and leg press exercises at moderate intensity (60% of 1‐RM) using continuous breathing and a modified BH technique without closed glottis, stating that the hemodynamic response is highly dependent on the execution technique.

It is noteworthy that studies on this topic have primarily been conducted with male participants, focusing mainly on basic cardiopulmonary parameters (MacDougall et al., 1985; Narloch & Brandstater, 1995). However, due to known sex‐based physiological differences in cardiovascular control and cardiorespiratory function – such as lower arterial stiffness, different autonomic regulation, and reduced SV and cardiac output responses in women (Ansdell et al., 2020; Fu & Ogoh, 2019; Joyner et al., 2016; Lasocka‐Koriat et al., 2024; Lu & Yin, 2023; Saz‐Lara et al., 2025) – findings from male participants may not be generalizable to females. SV, cardiac output, and V̇O_2_ are underexplored in strength training, and no studies have examined the effects of breathing techniques in females. Moreover, women are systematically underrepresented in exercise physiology research, contributing to a persistent gender data gap in sports science and medicine (Cowley et al., 2024; Smith et al., 2022; Sonnier et al., 2024).

Another aspect that remains insufficiently explored is how the intensity of resistance exercise modulates these physiological responses. Since the magnitude of intra‐abdominal and intrathoracic pressure increases with higher workloads, exercise intensity is expected to amplify the hemodynamic effects of BH or VM (MacDougall et al., 1985; Narloch & Brandstater, 1995). Investigating different intensities can therefore help determine the threshold at which these responses become significant or clinically relevant.

Post‐exercise hypotension refers to the acute reduction in blood pressure observed after resistance exercise (de Oliveira Carpes et al., 2021; Duncan et al., 2014; Rezk et al., 2006; Tibana et al., 2014). This response, typically lasting several minutes to 24 h, is attributed to mechanisms such as sustained peripheral vasodilation and altered autonomic regulation (Farinatti et al., 2021; MacDonald, 2002). Because some cardiovascular effects become apparent after exercise cessation, post‐exercise assessments allow a more comprehensive evaluation of hemodynamic adjustments. Moreover, exercise intensity and breathing techniques can influence acute cardiovascular responses during resistance exercise and may thus modulate the magnitude of hypotension (Duncan et al., 2014; MacDonald, 2002; Rezk et al., 2006).

Consequently, this randomized, crossover study aimed to analyze acute hemodynamic and cardiopulmonary responses during and after strength training, both with and without BH, at various intensities in young, healthy, physically active women. Based on prior evidence, we expected the strongest cardiovascular and respiratory responses to BH at higher exercise intensities.

## 2 MATERIALS AND METHODS

### 2.1 Ethical approval

Our study adhered to the latest version of the Declaration of Helsinki and received approval from the Ethics Committee of the Medical Faculty at the University of Leipzig (approval number: 023/23‐ek).

### 2.2 Participants

The sample included 17 healthy female subjects with at least 1 year of experience in strength training (Table 1). These individuals had no cardiac, pulmonary, inflammatory diseases, or orthopedic issues at the time of the examinations. Participants were required to be technically and physically capable of completing the strength training tasks. Written informed consent was obtained from all participants.

**TABLE 1:** Baseline characteristics of the study participants (*n* = 17).

Anthropometric and physical parameters	
Age (years)	22.8 ± 1.9
Height (cm)	166.0 ± 5.6
Weight (kg)	60.6 ± 6.9
BMI (kg/m^2^)	22.0 ± 2.4
LBM (kg)	46.5 ± 3.4
FM (%)	23.5 ± 5.4
Physical activity (hours per week)	7.35 ± 1.93
10‐RM (kg)	59.9 ± 7.3
IET outcomes at *P*_max_	
*P*_max_ IET (W)	205.3 ± 29.5
Relative *P*_max_ IET (W/kg)	3.41 ± 0.51
HR (bpm)	185.4 ± 6.8
SV (mL)	92.6 ± 20.3
CO (L/min)	15.45 ± 2.97
SBP (mmHg)	185.1 ± 13.7
RPP	34,377 ± 3102
RR (bpm)	45.1 ± 8.1
V_T_ (L)	2.11 ± 0.19
V̇_E_ (L/min)	94.60 ± 16.01
V̇O_2_ (mL/min/kg)	41.5 ± 5.5
Lac (mmol/L)	10.7 ± 2.2
RPE (0–10)	9.94 ± 0.24

*Note*: Values are presented as means and standard deviations.

Abbreviations: 10‐RM, 10‐repetition‐maximum; BMI, body mass index; CO, cardiac output; FM, fat mass; HR, heart rate; IET, incremental exercise test; Lac, blood lactate; LBM, lean body mass; *P*_max_, maximum power output; RPE, rating of perceived exertion (BORG 0–10); RPP, rate‐pressure product; RR, respiratory rate; SBP, systolic blood pressure; SV, stroke volume; V̇_E_, minute ventilation; V̇O_2_, oxygen uptake; V_T_, tidal volume.

Data from Chigira et al. (2024) indicate that BH (VM) during squats increased SBP by 7 mmHg compared to squats without BH, with a pooled standard deviation of 12. Given the within‐study design, we assumed a correlation of 0.7 between repeated measurements, yielding a Cohen's *d* of 0.75. To achieve a statistical power of 0.8 with an alpha level of 0.05, a minimum of 16 participants needed to be included in the study. Notably, the blood pressure values reported by Chigira et al. (2024) were obtained post‐exercise. Measurements during exertion were expected to yield greater effects.

### 2.3 Study design

**FIGURE 1:**
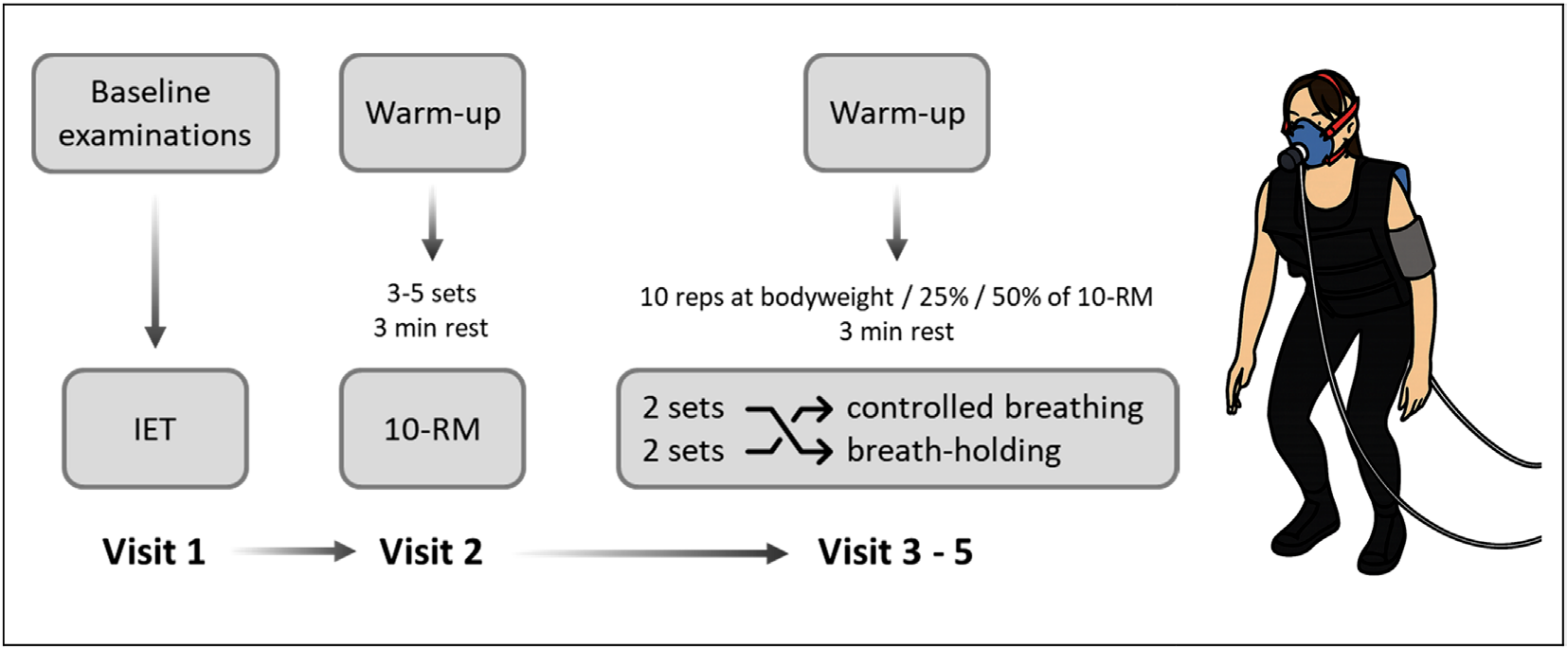
Flow chart depicting the study protocol. At visit 1, baseline examinations and the incremental exertion test (IET) were performed to ensure individual health and physical conditions. The individual's 10‐repetition maximum (10‐RM) of squats with a weight vest was determined at visit 2. The three conditions (4 sets of 10 repetitions (reps) with bodyweight, at 25% or 50% of 10‐RM, with a resting period of 3 min between sets) were performed on separate days. Breathing techniques included 2 sets with controlled breathing and 2 sets with breath‐holding. The order of visits 3–5 and the order of the breathing techniques were randomized.

The study comprised five visits (Figure 1). Following the initial screening (visit 1) and the 10‐repetition maximum (RM) test (visit 2), all participants completed three randomized training sessions of squats with and without BH (visits 3–5) and at different intensities (bodyweight, 25%, and 50% of 10‐RM additional weight). There were at least 48 h of exercise‐free periods between all study visits to ensure adequate regeneration.

During the **preliminary examinations** (visit 1), participants' physical health and suitability were assessed using their medical history, a lifestyle questionnaire (which included questions on physical activity, smoking, and alcohol consumption), an incremental exercise test, a lung function test conducted with the Easy on PC from ndd Medizintechnik AG (Zürich, Switzerland), echocardiography (using the GE Vivid E95, GE Healthcare GmbH, Düsseldorf, Germany), and bioelectrical impedance analysis with the Bioimpedance Analyzer BIACORPUS RX 4004 M (MEDI CAL HealthCare GmbH, Karlsruhe, Germany).

The exercise testing was performed on a semi‐recumbent ergometer (Ergoselect, ergoline GmbH, Bitz, Germany) at a standard cadence of 60–70 rpm to determine the maximum power output, as well as cardiac and pulmonary maximum values, e.g., maximum oxygen uptake. It started at 50 watts with increments of 15 watts per minute until exhaustion, defined by a cadence below 60 rpm, a respiratory exchange ratio above 1.1, and/or reaching the limit of perceived exertion of 10 on Borg's scale (Borg et al., 1985). Subsequently, the test was continued for a five‐minute recovery period at 25% of the individual's maximum power output. Electrocardiography, cardiopulmonary, and metabolic values were continuously measured.

After standardized warm‐up, all participants completed a **10‐RM test** (visit 2) following the guidelines for 1‐RM (Ozemek et al., 2025). Squats with a weight vest were performed using a standard procedure, with feet hip‐width apart and maximum knee flexion achieved when the femurs were parallel to the floor. The squat movement was controlled over three phases: eccentric, concentric, and upright stance. Each phase lasted 2 s, resulting in a movement speed of 6 s per repetition. If the test subject completed more than 10 repetitions according to the specified execution, the set was aborted and repeated after a 3‐min break with additional weight. In case of an incomplete attempt (< 10 repetitions), the previous load was assumed to be 10‐RM weight.

The main study part consisted of three **strength training sessions** (visits 3–5). After a 15‐min standardized warm‐up consisting of bicycle ergometer and mobilization exercises, the test subjects were equipped with the measuring devices, and then they performed the strength training sessions. There were at least 48 h between strength training sessions without physical exercise. In a block‐randomized order, the subjects completed four sets of ten repetitions of free‐weight squats, either without additional weight or using a weight vest loaded with 25% or 50% of their 10‐RM. In each session, two consecutive sets were completed in randomized order with controlled breathing (CB) or with breath‐holding (BH).

The squat movement was performed identically to the 10‐RM test, consisting of a two‐second eccentric phase (descending), a two‐second concentric phase (ascending), and a two‐second standing phase (upright), for a total of 6 s per squat and 60 s per set. A three‐minute rest was provided between sets to ensure adequate cardiopulmonary and metabolic recovery (de Salles et al., 2009). This extended set duration, which includes an additional stance phase, was selected because it provides a higher measurement resolution than the typical four‐second duration and allows for a technically interference‐free manual blood pressure measurement. The squat's range of motion was standardized as described in the 10‐RM test. The participants received feedback from the test administrator on the correct execution of the exercises. In the CB condition, the subjects were instructed to exhale continuously and audibly during the concentric and standing phase of the squat (4 s). In contrast, in the BH condition, the subjects had to hold their breath during the concentric phase and press against the closed glottis (2 s), as in the Valsalva maneuver (VM), before exhaling during the stance phase (2 s) and inhaling again during the eccentric phase (2 s). A visual and acoustic signal accompanied the squatting and breathing phases. We observed the execution of the BH by audible respiratory sounds and the recorded inspiratory and expiratory times. Since the spiroergometry system determines exhalation time only from the turbine signal (the duration of turbine rotation), the software calculates inhalation time as the interval between two consecutive exhalations. Consequently, the duration of BH following inhalation cannot be quantified separately and is inherently included in the calculated inhalation time. As we monitored the inhalation acoustically during testing, the differences in inhalation time between the CB and BH conditions can reasonably be interpreted as reflecting the duration of BH. However, intrathoracic and abdominal pressures were not measured, and mechanical verification of glottal closure was precluded by the spirometry mask. During the rest periods before and after the sets, participants sat in a chair and stood up 30 s before each set began.

### 2.4 Measurements during incremental exercise test and strength training sessions

Electrocardiography (custo cardio BT‐A 202300, custo med GmbH, Ottobrunn, Germany), impedance cardiography (PhysioFlow PF07 Q‐Link, Manatec Biomedical, Petit Ebersviller, France), and spiroergometry (Metalyzer 3B, Cortex Biophysik GmbH, Leipzig, Germany) were used for continuous monitoring. The hemodynamic parameters heart rate (HR), stroke volume (SV), cardiac output (CO), and the pulmonary parameters respiratory rate, tidal volume, minute ventilation (V̇_E_), and oxygen uptake (V̇O_2_) were examined. The electrodes for impedance measurement were applied in a standardized manner after skin preparation (exfoliation and disinfection) in accordance with the manufacturer's instructions for each training session. No signal loss occurred during the exercises, minor drops in signal quality were excluded from the analysis. In addition, we registered arterial systolic blood pressure (SBP) per manual measurement (auscultatory method according to Riva‐Rocci and Korotkoff), blood lactate level by capillary blood sampling (Super GL ambulance, Dr. Müller, Freital, Germany), and assessed the rating of perceived exertion using Borg's scale (Borg et al., 1985). SBP was measured during the last three repetitions of each set. The blood pressure cuff was inflated during the two previous repetitions (6th and 7th). The SBP was then determined exclusively during the concentric phases of the 8th to 10th repetitions by slowly deflating the cuff at approximately 5 mmHg per second. These intervals corresponded to either prescribed BH or continuous exhalation. Immediately after the set ended, lactate samples were taken, and perceived exertion was queried. All measurements were repeated after 1 min and after a 3‐min break.

For further analysis, all measurement data, excluding arterial blood pressure values, were initially averaged to 10‐s intervals. In addition, mean values for two sets of squats (2 × 60 s) and peak values for the last five repetitions (30 s) were generated separately for each breathing technique. We also averaged the data from the first minute of each recovery phase (2 × 60 s) separately for each breathing technique. The rate‐pressure product was calculated using the following formula: HR × SBP (Hui et al., 2000).

### 2.5 Statistical analysis

All data were processed using Microsoft Excel 2010 (Microsoft Corporation, Redmond, WA, USA) and GraphPad Prism 10 (GraphPad Software, Boston, MA, USA) for Windows. Unless otherwise stated, the tables show mean values and standard deviations. Normality was assessed using the Shapiro–Wilk test. If data were normally distributed, the statistical analysis was performed using a two‐way repeated‐measures analysis of variance (RM‐ANOVA) and Tukey's post hoc tests. The significance level of *p* < 0.05 applies to all results shown. The effect size, partial eta‐squared (*η* ^2^ *_p_*), for RM‐ANOVAs was rated according to the following benchmarks: small (*η* ^2^ *_p_* > 0.01), medium (*η* ^2^ *_p_* > 0.06), and large (*η* ^2^ *_p_* > 0.14) effects (Cohen, 1988). Some non‐normally distributed baseline values were examined using the Friedman test with Dunn‐Bonferroni post hoc analysis.

## 3 RESULTS

### 3.1 Performance and baseline characteristics of participants

The average peak values of the preliminary exercise tests are shown in Table 1. All results are given as mean values (± standard deviations).

There were significant baseline differences in SBP between the strength training sessions with different intensities (bodyweight: 116 (±7) mmHg vs. 25%: 116 (±8) mmHg vs. 50%: 120 (±10) mmHg; *p* = 0.0210). Further significant baseline differences occurred in respiratory rate (bodyweight: 17 (±3) bpm vs. 25%: 19 (±3) bpm vs. 50%: 20 (±2) bpm; *p* = 0.0037), inspiratory time (bodyweight: 1.48 (±0.34) seconds vs. 25%: 1.35 (±0.22) seconds vs. 50%: 1.28 (±0.19) seconds; *p* = 0.0144), and expiratory time (bodyweight: 2.56 (±0.71) seconds vs. 25%: 2.17 (±0.39) seconds vs. 50%: 2.03 (±0.33) seconds; *p* = 0.0022).

### 3.2 Cardiopulmonary outcomes during strength training sessions

Table 2 shows the mean peak values during the last five squats. The breathing pattern revealed significant differences across all parameters, except for SV and blood lactate. The largest main effects occurred in peak SBP, rate‐pressure product, V̇_E_, V̇O_2_, inspiratory and expiratory time, and rating of perceived exertion. Inspiratory time (2 s eccentric phase and BH during concentric phase) was twice as long, while expiratory time was significantly shorter during the application of BH, confirming the execution of the BH. Only SV, cardiac output, and tidal volume showed no main differences in intensity. Furthermore, the respiratory rate and tidal volume showed an interaction effect: BH maintained a consistent respiration rate and tidal volume across all intensities, while CB exhibited an intensity‐dependent variation in both respiration rate and tidal volume.

**TABLE 2:** Peak values during exercise period (*n* = 17, two sets of 10 repetitions with controlled breathing and two sets with breath‐holding, excluding rest periods).

	Intensity	Breathing pattern		*p*‐value (effect size *η* ^2^ *_p_*)		
		Controlled breathing	Breath‐holding	Breathing pattern	Intensity	Interaction
*Hemodynamic parameters*						
HR (bpm)	**Bodyweight**	**99.54 ± 15.53** ^a^, ^c^, ^d^	**104.20 ± 20.31** ^a^, ^d^	**0.0053** (0.393)	**<0.0001** (0.543)	0.3826 (0.056)
	**25%**	**107.07 ± 14.90** ^a^, ^b^, ^d^	**109.63 ± 16.50** ^a^, ^d^			
	**50%**	**115.76 ± 19.04** ^a^, ^b^, ^c^	**119.41 ± 19.22** ^a^, ^b^, ^c^			
SV (mL)	Bodyweight	64.93 ± 14.57	66.34 ± 12.62	0.0897 (0.169)	0.8491 (0.010)	0.4980 (0.039)
	25%	66.79 ± 13.41	67.18 ± 14.13			
	50%	66.14 ± 10.73	69.35 ± 13.82			
CO (L/min)	**Bodyweight**	**6.48 ± 1.89** ^a^	**6.95 ± 2.08** ^a^	**0.0227** (0.284)	0.0669 (0.161)	0.4367 (0.046)
	25%	7.16 ± 1.93	7.40 ± 2.26			
	50%	7.55 ± 1.36	8.18 ± 1.85			
SBP (mmHg)	**Bodyweight**	**140.5 ± 15.1** ^a^, ^c^, ^d^	**173.4 ± 19.2** ^a^, ^d^	**<0.0001** (0.921)	**<0.0001** (0.618)	0.0825 (0.157)
	**25%**	**148.6 ± 13.2** ^a^, ^b^, ^d^	**181.0 ± 14.9** ^a^, ^d^			
	**50%**	**162.3 ± 10.8** ^a^, ^b^, ^c^	**189.1 ± 16.3** ^a^, ^b^, ^c^			
RPP	**Bodyweight**	**14,163 ± 3684** ^a^, ^c^, ^d^	**18,351 ± 5458** ^a^, ^d^	**<0.0001** (0.829)	**<0.0001** (0.608)	0.6767 (0.018)
	**25%**	**16,017 ± 3234** ^a^, ^b^, ^d^	**19,959 ± 4021** ^a^, ^d^			
	**50%**	**18,905 ± 3913** ^a^, ^b^, ^c^	**22,713 ± 4864** ^a^, ^b^, ^c^			
*Pulmonary and metabolic parameters*						
RR (bpm)	bodyweight	10.79 ± 2.01^d^	10.19 ± 1.08	**0.0075** (0.369)	**0.0238** (0.263)	**0.0081** (0.342)
	25%	11.15 ± 2.82	10.18 ± 0.77			
	**50%**	**14.59 ± 5.55** ^a^, ^b^	**10.37 ± 1.30** ^a^			
V_T_ (L)	**Bodyweight**	**2.343 ± 0.570** ^a^	**1.970 ± 0.484** ^a^	**0.0385** (0.241)	0.4525 (0.042)	**0.0158** (0.266)
	**25%**	**2.368 ± 0.635** ^a^	**2.084 ± 0.421** ^a^			
	50%	2.095 ± 0.615	2.145 ± 0.388			
V̇_E_ (L/min)	**Bodyweight**	**24.13 ± 4.42** ^a^	**19.96 ± 5.12** ^a^, ^d^	**<0.0001** (0.712)	**0.0081** (0.270)	0.7480 (0.016)
	**25%**	**24.82 ± 5.04** ^a^	**20.93 ± 3.87** ^a^			
	**50%**	**26.49 ± 3.60** ^a^	**21.94 ± 4.02** ^a^, ^b^			
TI (s)	**Bodyweight**	**2.27 ± 0.31** ^a^	**4.25 ± 0.55** ^a^	**<0.0001** (0.922)	**0.0465** (0.202)	0.8438 (0.008)
	**25%**	**2.25 ± 0.35** ^a^	**4.18 ± 0.47** ^a^			
	**50%**	**2.05 ± 0.46** ^a^	**3.99 ± 0.80** ^a^			
TE (s)	**Bodyweight**	**3.55 ± 0.46** ^a^, ^d^	**1.81 ± 0.41** ^a^	**<0.0001** (0.849)	**0.0401** (0.220)	**0.0011** (0.425)
	**25%**	**3.43 ± 0.63** ^a^, ^d^	**1.82 ± 0.46** ^a^			
	**50%**	**2.90 ± 0.94** ^a^, ^b^, ^c^	**1.92 ± 0.73** ^a^			
V̇O_2_ (L/min)	**Bodyweight**	**0.750 ± 0.117** ^a^, ^c^, ^d^	**0.656 ± 0.126** ^a^, ^c^, ^d^	**<0.0001** (0.663)	**<0.0001** (0.879)	0.1010 (0.141)
	**25%**	**0.837 ± 0.127** ^a^, ^b^, ^d^	**0.744 ± 0.084** ^a^, ^b^, ^d^			
	**50%**	**0.982 ± 0.142** ^a^, ^b^, ^c^	**0.835 ± 0.103** ^a^, ^b^, ^c^			
V̇CO_2_ (L/min)	**Bodyweight**	**0.732 ± 0.128** ^a^, ^d^	**0.633 ± 0.122** ^a^, ^c^, ^d^	**0.0002** (0.581)	**<0.0001** (0.498)	0.5704 (0.030)
	**25%**	**0.763 ± 0.133** ^a^, ^d^	**0.675 ± 0.089** ^a^, ^b^			
	**50%**	**0.842 ± 0.131** ^a^, ^b^, ^c^	**0.728 ± 0.092** ^a^, ^b^			
Lac (mmol/L)	Bodyweight	1.10 ± 0.54^d^	1.17 ± 0.70^d^	0.1753 (0.112)	**0.0021** (0.393)	0.5976 (0.022)
	25%	1.36 ± 0.61	1.40 ± 0.65^d^			
	50%	1.67 ± 0.89^b^	1.87 ± 1.17^b^, ^c^			
RPE	Bodyweight	2.41 ± 0.69^d^	2.74 ± 0.77^d^	**0.0023** (0.449)	**<0.0001** (0.658)	0.3650 (0.060)
	**25%**	**2.76 ± 0.94** ^a^, ^d^	**3.29 ± 0.75** ^a^, ^d^			
	**50%**	**3.94 ± 1.18** ^a^, ^b^, ^c^	**4.68 ± 0.95** ^a^, ^b^, ^c^			

*Note*: Values are presented as means and standard deviations; Bold values indicate significant Post‐hoc‐tests (*p* < 0.05).

Abbreviations: 10‐RM, 10‐repetition‐maximum; CO, cardiac output; HR, heart rate; Lac, blood lactate; RPE, rating of perceived exertion (BORG 0–10); RPP, rate‐pressure product; RR, respiratory rate; SBP, systolic blood pressure; SV, stroke volume; TE, expiratory time; TI, inspiratory time; V̇CO_2_, carbon dioxide output; V̇_E_, minute ventilation; V̇O_2_, oxygen uptake; V_T_, tidal volume; *η* ^2^ *_p_*, partial eta‐squared of the two‐way repeated measures ANOVA.

^a^ Different in breathing pattern (controlled breathing vs. breath‐holding) (*p* < 0.05).

^b^ Different from bodyweight (*p* < 0.05).

^c^ Different from 25% of 10‐RM (*p* < 0.05).

^d^ Different from 50% of 10‐RM (*p* < 0.05).

**FIGURE 2:**
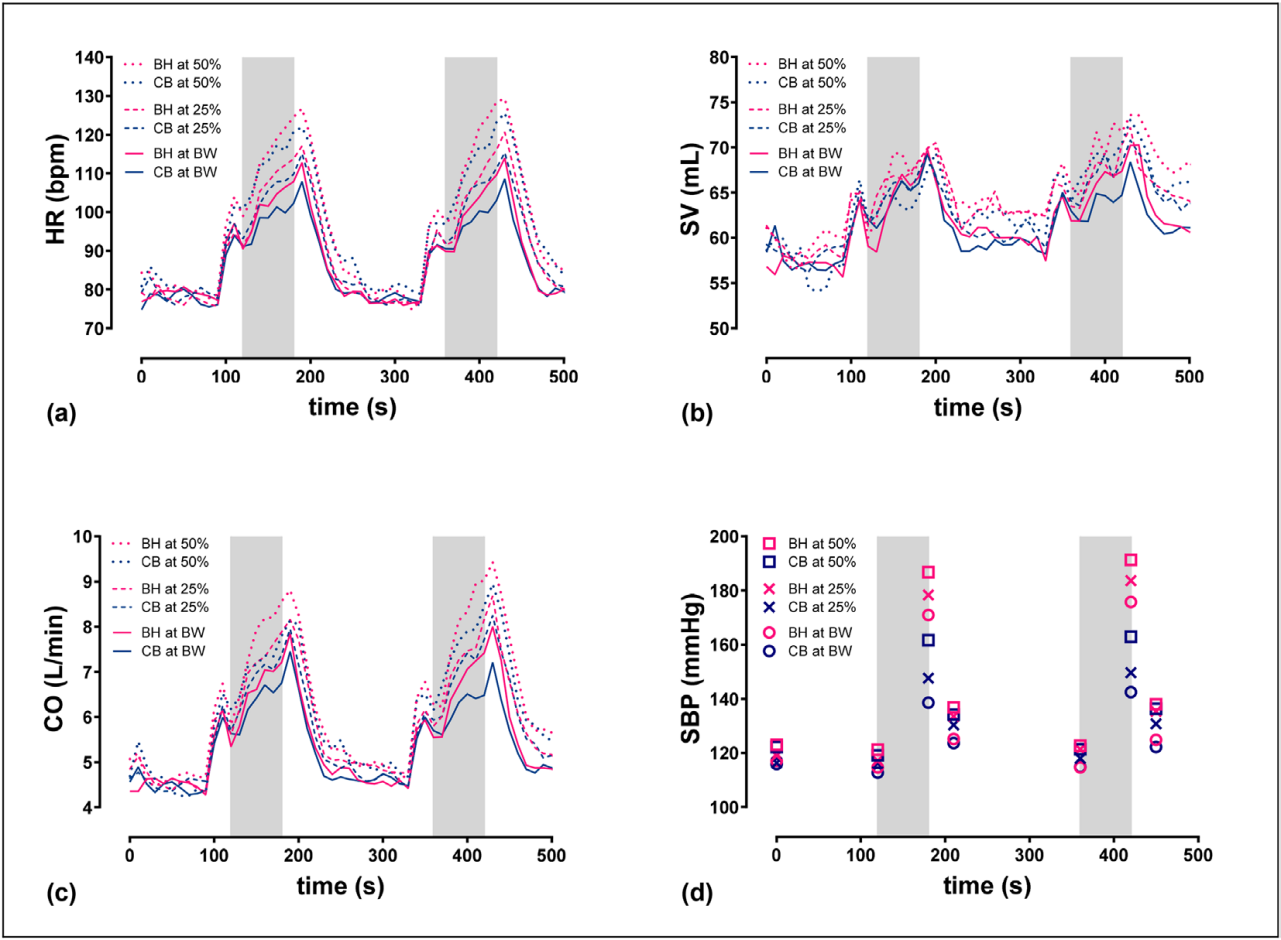
Graphs show the mean hemodynamic response during strength training sessions with bodyweight, at 25% and 50% of 10‐repetition maximum (*n* = 17, two sets of ten repetitions). (a) heart rate (HR); (b) stroke volume (SV); (c) cardiac output (CO); (d) systolic blood pressure (SBP); Gray‐shaded areas indicate the exercise intervals of the strength training sessions; exercise intervals lasted 60 s for 10 repetitions, resting periods between all sets were 3 min; BH, breath‐holding; BW, bodyweight; CB, controlled breathing.

During the test, participants' HR increased abruptly upon standing before the sets, then steadily during the squats (Figure 2a). SV similarly reflects the transition from sitting to standing prior to set initiation, followed by a non‐continuous rise during the set across all conditions, with a slight decline toward the end of the exercise period. During recovery, SV exhibits a brief, pronounced increase before rapidly returning to baseline levels (Figure 2b). The blood pressure graphs are derived from individual measurements taken before, during, and after the exercise periods. All blood pressure values during the BH squats are higher than those during CB (Figure 2d).

When exercising with body weight, BH resulted in 149% of the resting SBP, while controlled breathing (CB) elicited 122% of the resting SBP. With an additional load, which corresponded to 50% of 10‐RM, SBP achieved 159% of the resting values with HB versus 136% at the CB condition.

**FIGURE 3:**
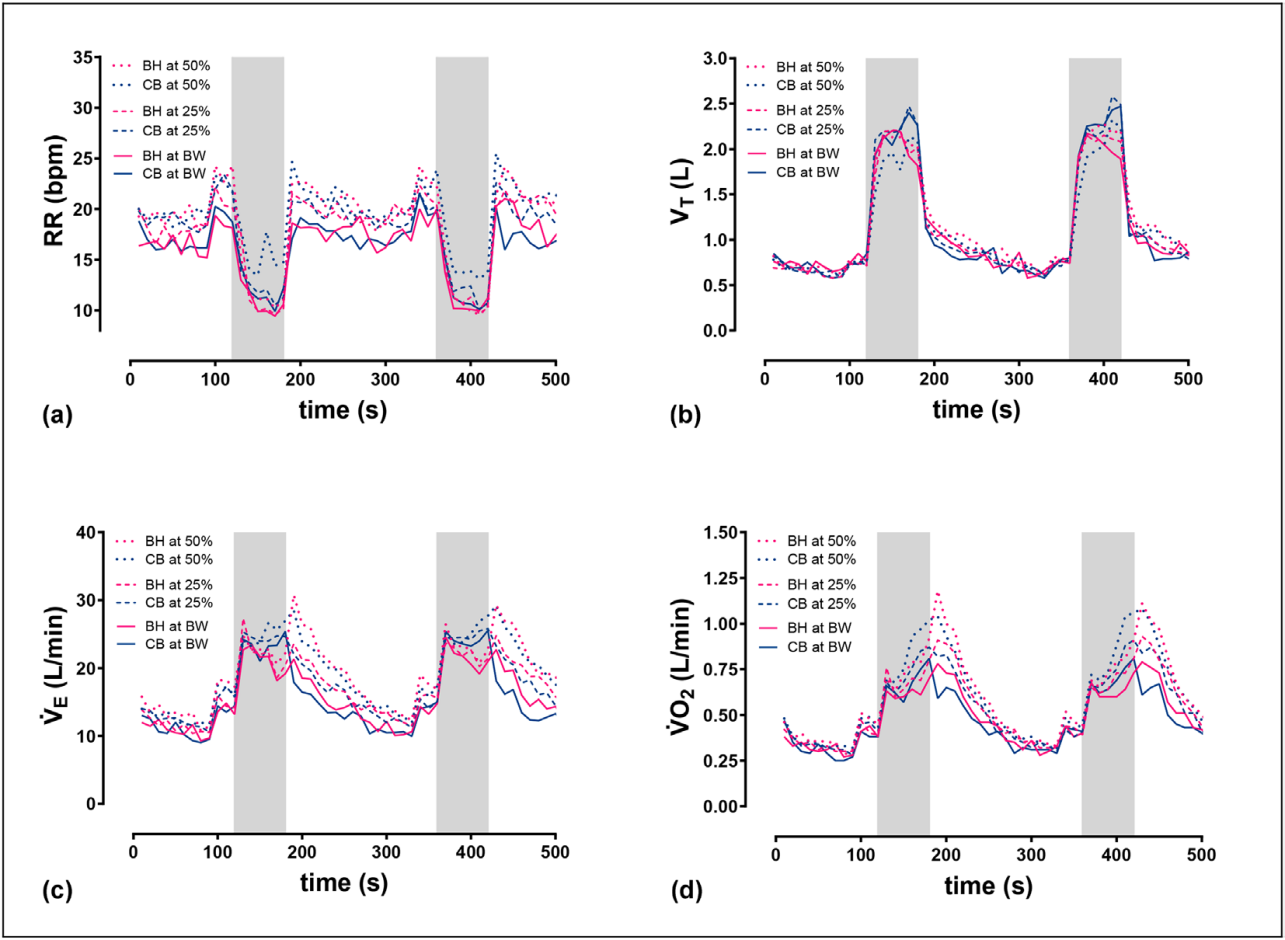
Graphs show the mean pulmonary response during strength training sessions with bodyweight, 25% and 50% of 10‐repetition maximum (*n* = 17, two sets of ten repetitions). (a) respiratory rate (RR); (b) tidal volume (V_T_); (c) minute ventilation (V̇_E_); (d) oxygen uptake (V̇O_2_); Gray‐shaded areas indicate the exercise intervals of the strength training sessions; exercise intervals lasted 60 s for 10 repetitions, resting periods between all sets were 3 min; BH, breath‐holding; BW, bodyweight; CB, controlled breathing.

Compared with the resting periods, the respiratory rate decreased markedly during the squats, while tidal volume increased reciprocally (Figure 3a,b). Tidal volume and V̇_E_ decreased during BH sets and increased rapidly at the end of the exercise, whereas the opposite was observed during CB (Figure 3b,c). V̇O_2_ increased continuously throughout the sets in both conditions, but decreased more slowly during the recovery periods after the squats with BH than with CB (Figure 3d).

With BH, participants reached 21%–24% of maximum V̇_E_. In contrast, the peak values for CB ranged from 26% to 29% of maximum V̇_E_. V̇O_2_ is also significantly reduced by BH, regardless of intensity. Peak V̇O_2_ during exercise with BH ranged from 27% to 34%, compared with 30% to 40% of maximum V̇O_2_ under CB. The BH intervals were characterized by notably longer inhalation and shorter exhalation durations during the BH condition (Table 2).

### 3.3 Cardiopulmonary outcomes in the post‐exercise period

Table 3 presents the results of the first minute of recovery. No interaction effects between the effects of breathing pattern and intensity were found in the recovery period. The breathing pattern had a significant main effect on HR and its dependent parameters, cardiac output, and rate‐pressure product, post‐exercise. For the pulmonary parameters, only tidal volume, V̇_E_, and V̇O_2_ showed significantly higher recovery values in the BH condition. We observed large effect sizes across all significant differences. All recorded parameters, except SV, showed a substantial dependence on intensity.

**TABLE 3:** Post‐exercise values during rest period (*n* = 17, 60 s post‐exercise).

	Intensity	Breathing pattern		*p*‐value (effect size *η* ^2^ *_p_*)		
		Controlled breathing	Breath‐holding	Breathing pattern	Intensity	Interaction
*Hemodynamic parameters*						
HR (bpm)	Bodyweight	94.25 ± 15.68^c^, ^d^	96.95 ± 19.30^c^, ^d^	**0.0055** (0.392)	**0.0003** (0.487)	0.9484 (0.003)
	**25%**	**101.37 ± 16.09** ^a^, ^b^, ^d^	**103.54 ± 14.66** ^a^, ^b^, ^d^			
	50%	110.18 ± 20.00^b^, ^c^	112.33 ± 19.67^b^, ^c^			
SV (mL)	Bodyweight	63.91 ± 13.87	65.21 ± 13.64	0.1129 (0.150)	0.6420 (0.027)	0.7941 (0.014)
	25%	66.58 ± 13.70	67.35 ± 14.74			
	50%	66.89 ± 12.26	69.11 ± 12.96			
CO (L/min)	**Bodyweight**	**6.04 ± 1.63** ^a^, ^d^	**6.38 ± 1.94** ^a^	**0.0264** (0.272)	**0.0271** (0.210)	0.8748 (0.007)
	25%	6.76 ± 1.82	7.00 ± 1.94			
	50%	7.28 ± 1.31^b^	7.68 ± 1.52			
SBP (mmHg)	Bodyweight	122.9 ± 15.8^c^, ^d^	125.0 ± 18.6^d^	0.0826 (0.230)	**0.0017** (0.418)	0.6923 (0.028)
	25%	130.5 ± 9.9^b^	133.8 ± 11.0			
	50%	135.7 ± 13.3^b^	137.4 ± 17.0^b^			
RPP	Bodyweight	11,922 ± 3381^c^, ^d^	12,589 ± 4629	**0.0038** (0.516)	**0.0319** (0.289)	0.9790 (0.001)
	**25%**	**13,233 ± 2947** ^a^, ^b^	**13,966 ± 3291** ^a^			
	50%	14,524 ± 3720^b^	15,221 ± 4354			
*Pulmonary and metabolic parameters*						
RR (bpm)	Bodyweight	17.75 ± 3.45^c^, ^d^	18.74 ± 2.85^d^	0.2958 (0.068)	**0.0002** (0.442)	0.1849 (0.101)
	25%	20.33 ± 2.60^b^	20.60 ± 2.99			
	50%	22.18 ± 2.92^b^	21.72 ± 2.89^b^			
V_T_ (L)	**Bodyweight**	**0.904 ± 0.219** ^a^, ^d^	**0.985 ± 0.264** ^a^, ^d^	**0.0005** (0.544)	**0.0022** (0.369)	0.4798 (0.041)
	**25%**	**0.969 ± 0.201** ^a^, ^d^	**1.016 ± 0.223** ^a^, ^d^			
	**50%**	**1.074 ± 0.196** ^a^, ^b^, ^c^	**1.142 ± 0.229** ^a^, ^b^, ^c^			
V̇_E_ (L/min)	**Bodyweight**	**15.11 ± 3.26** ^a^, ^c^, ^d^	**17.56 ± 3.50** ^a^, ^c^, ^d^	**0.0001** (0.618)	**<0.0001** (0.840)	0.1132 (0.128)
	**25%**	**19.25 ± 4.05** ^a^, ^b^, ^d^	**20.40 ± 4.19** ^a^, ^b^, ^d^			
	50%	23.36 ± 4.71^b^, ^c^	24.44 ± 5.23^b^, ^c^			
TI (s)	Bodyweight	1.48 ± 0.26^c^, ^d^	1.55 ± 0.31^c^, ^d^	**0.0202** (0.294)	**0.0007** (0.379)	0.8714 (0.008)
	25%	1.32 ± 0.16^b^	1.38 ± 0.17^b^			
	50%	1.27 ± 0.23^b^	1.31 ± 0.22^b^			
TE (s)	**Bodyweight**	**2.38 ± 0.55** ^a^, ^c^, ^d^	**2.13 ± 0.62** ^a^, ^d^	**0.0001** (0.605)	**0.0004** (0.459)	0.0792 (0.155)
	25%	1.92 ± 0.31^b^	1.82 ± 0.30			
	50%	1.74 ± 0.34^b^	1.71 ± 0.35^b^			
V̇O_2_ (L/min)	**Bodyweight**	**0.555 ± 0.104** ^a^, ^c^, ^d^	**0.640 ± 0.109** ^a^, ^c^, ^d^	**<0.0001** (0.724)	**<0.0001** (0.893)	0.1402 (0.118)
	**25%**	**0.707 ± 0.109** ^a^, ^b^, ^d^	**0.753 ± 0.113** ^a^, ^b^, ^d^			
	**50%**	**0.817 ± 0.088** ^a^, ^b^, ^c^	**0.865 ± 0.096** ^a^, ^b^, ^c^			
V̇CO_2_ (L/min)	**Bodyweight**	**0.442 ± 0.097** ^a^, ^c^, ^d^	**0.524 ± 0.118** ^a^, ^c^, ^d^	**<0.0001** (0.692)	**<0.0001** (0.799)	0.1323 (0.124)
	**25%**	**0.563 ± 0.118** ^a^, ^b^, ^d^	**0.610 ± 0.126** ^a^, ^b^, ^d^			
	**50%**	**0.692 ± 0.130** ^a^, ^b^, ^c^	**0.735 ± 0.139** ^a^, ^b^, ^c^			

*Note*: Values are presented as means and standard deviations; Bold values indicate significant Post‐hoc‐tests (*p* < 0.05).

Abbreviations: 10‐RM, 10‐repetition‐maximum; CO, cardiac output; HR, heart rate; RPP, rate‐pressure product; RR, respiratory rate; SBP, systolic blood pressure; SV, stroke volume; TE, expiratory time; TI, inspiratory time; V̇CO_2_, carbon dioxide output; V̇_E_, minute ventilation; V̇O_2_, oxygen uptake; V_T_, tidal volume; η^2^_p_, partial eta‐squared of the two‐way repeated measures ANOVA.

^a^ Different in breathing pattern (controlled breathing vs. breath‐holding) (*p* < 0.05).

^b^ Different from bodyweight (*p* < 0.05).

^c^ Different from 25% (*p* < 0.05).

^d^ Different from 50% of 10‐RM (*p* < 0.05).

## 4 DISCUSSION

The main findings of this randomized crossover study with continuous cardiopulmonary assessment in healthy women demonstrate that both cardiovascular and ventilatory responses during and after low‐to‐moderate‐intensity exercise were dependent on the breathing technique employed. Regardless of the applied intensity, breath‐holding (BH), confirmed by altered breathing patterns, compared to controlled breathing (CB) led to significantly higher HR and blood pressure values. V̇_E_, V̇O_2_, and carbon dioxide output were significantly reduced during exercise, as expected, while lactate values showed no significant differences. Post‐exercise, HR and cardiac output of healthy women revealed a slight but sustained increase after BH, accompanied by a compensatory ventilatory response. These findings collectively indicate that BH imposes a transient hemodynamic load while constraining ventilation during exercise, producing a coordinated cardiopulmonary adjustment that extends into recovery.

The reported differences in baseline measurements between intensity sessions are minor, likely due to the weight vest, and remain within normal physiological ranges. No baseline differences were observed between breathing conditions.

### 4.1 Hemodynamic mechanisms during breath‐holding

In our female participants, BH during strength training sessions with body weight or low additional loads significantly increased cardiac load, independent of intensity, with the strongest effects on HR, SBP, and rate‐pressure product. Even though the differences in HR and cardiac output are physiologically minor and may be clinically irrelevant, this pattern reflects the combined mechanical and reflex responses triggered by transient elevations in intrathoracic and intra‐abdominal pressure.

**FIGURE 4:**
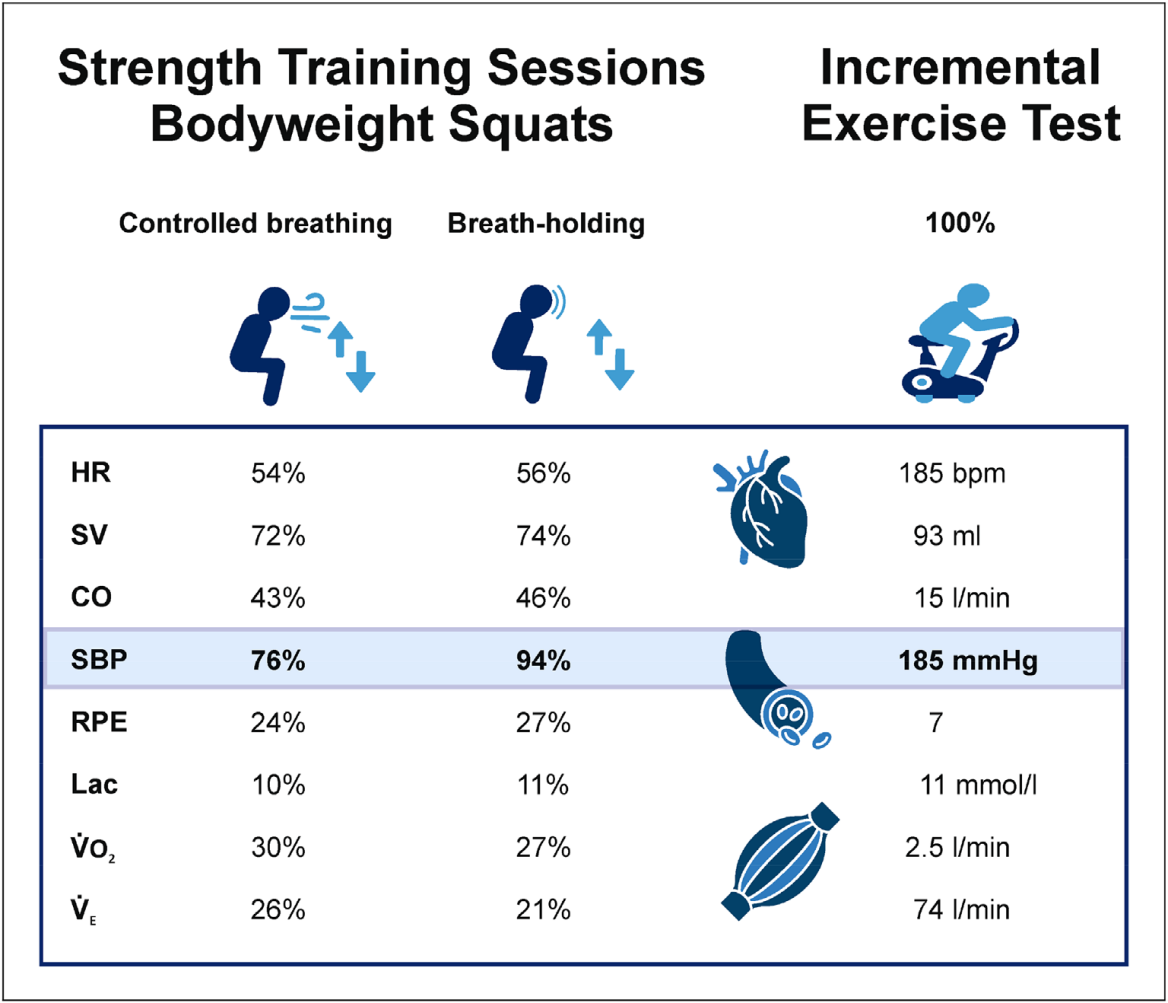
The diagram illustrates the acute cardiac, vascular, and metabolic responses to training sessions. These data correspond to the 100% values in an incremental exercise test on an ergometer. During breath‐holding, there was a significant increase in systolic blood pressure (SBP), reaching levels comparable to those observed during the incremental exercise test (IET). However, heart rate (HR), stroke volume (SV), and cardiac output (CO) showed only slight differences between the conditions and were considerably lower than those seen in IET. Additionally, the pulmonary response during breath‐holding was less pronounced compared to controlled breathing. Lac, blood lactate level; RPE, rating of perceived exertion (BORG 0–10); V̇_E_, minute ventilation; V̇O_2_, oxygen uptake.

Alongside previous studies with male athletes, SBP rose significantly higher during BH than during continuous exhalation (MacDougall et al., 1992; Narloch & Brandstater, 1995). Moreover, the shorter the expiratory time following BH, the greater the increase in SBP, suggesting that the duration of pressure release is a key determinant of the cardiovascular response. Performing squats at a comparable intensity (20% of 1‐RM) without prescribing a breathing technique, the SBP of female subjects increased to 121% of their resting value (Park, 2025), supporting our results under CB conditions. Though the peak values of HR and SBP under BH conditions did not reach those reported by other authors (Haykowsky et al., 2001; MacDougall et al., 1985; Narloch & Brandstater, 1995) – presumably due to the lower exercise intensity – the peak SBP still approached the maximum values observed during the incremental exercise test. Even during bodyweight squats, BH reached blood pressure levels 94% of the maximum (Figure 4). Nevertheless, according to ACSM's guidelines, the measured peak SBP values are not considered critical for healthy individuals (Ozemek et al., 2025).

In a study of 10 back squat repetitions at a higher intensity (50% of 3‐RM), male participants reached 81% of their maximum SBP without BH (Lässing, Hummelmann, et al., 2025). This emphasizes that the breathing pattern alone can significantly modulate cardiovascular stress, even when external load remains constant. Moreover, BH resulted in approximately a 20%–30% higher peak rate‐pressure product than CB, demonstrating that cardiac workload is significantly influenced by breathing pattern rather than exercise intensity per se.

The observed blood pressure rise during BH in our experimental setting can be mechanistically explained by several interrelated processes of force generation by abdominal muscle contraction and a closed glottis (Narloch & Brandstater, 1995). First, the performance of the VM, such as BH during squats, is associated with increased intrathoracic and intra‐abdominal pressures, elevating central vascular pressure (Blazek et al., 2019). Second, muscle contraction during squatting increases mechanical compression of the vasculature, thereby elevating total peripheral resistance. Mechanical and metabolic afferents activated during exercise (the exercise pressor reflex) stimulate sympathetic nervous system activity and, together with the reduced baroreflex sensitivity observed under load, substantially augment arterial blood pressure (Hartwich et al., 2011; Zhang et al., 2009). Particularly at lower intensities, the heart‐rate‐driven increase in cardiac output, combined with elevated pulmonary vascular resistance, results in markedly higher exercise blood pressure and consequently greater cardiac strain (Katayama & Saito, 2019; Teixeira & Vianna, 2022).

Before exercise onset, significant increases in cardiac and pulmonary parameters were observed, regardless of the breathing technique used (see Figures 2 and 3). This can be attributed to the change in body position from sitting to standing, which transiently alters venous return and baroreflex function (Lässing, Hochstein, et al., 2025). During the preparation of the measuring instruments, the test subjects sat, then stood before starting the squats. This dependence of the baroreflex on body position has been previously described under both resting and exercise conditions and is considered normal (Harms et al., 2021). During squat execution, we observed a pronounced initial drop in SV, followed by a limited increase during the repetitions, while HR increased linearly (Figure 2a,b). This trend was evident in both breathing patterns and across all intensities, indicating a consistent physiological adjustment rather than a group effect.

Some authors report a reduced venous return as a result of the VM, leading to a decreased SV (Delaney et al., 2020; Hughes et al., 1989; Mäntysaari et al., 1984; Narloch & Brandstater, 1995; Pstras et al., 2016), whereas Howard et al. (2018) detected no change in SV during high‐intensity squats with normal breathing. BH is also discussed in the context of diving, where distinct physiological regulatory mechanisms occur. When a person submerges underwater, the diving reflex is triggered, which is characterized by apnea, bradycardia, and increased peripheral vascular resistance (Godek & Freeman, 2025). However, Walsh et al. (2025) also reported a significantly higher HR when participants performed constant BH instead of breathing freely during high‐intensity ergometric exercise. A key difference from previous studies is the short duration and the repeated BH in our protocol (2 s during the concentric phase of the squat). Whereas a single, prolonged breath‐hold during exercise can reduce oxygen saturation and elicit various metabolic and physiological responses, short breath‐holds during resistance exercise repetitions are more likely to affect cardiovascular function than to substantially reduce oxygen saturation, as breathing continues throughout the exercise. In contrast to a single prolonged VM, repeated short BHs during strength exercise repetitions seem to impair venous return less and therefore do not inhibit the exercise‐induced increase in SV. Similarly, Hughes et al. (1989) found a minor decrease in SV during a VM combined with an isometric handgrip exercise than during a VM alone. It remains unclear whether the ratio of intrathoracic and intra‐abdominal pressure changes has a greater influence on the baroreflex than the total peripheral pressure (Gallagher et al., 2006; Katayama & Saito, 2019).

At the beginning of the rest interval, HR remained elevated following BH, while SBP equalized quickly across the different breathing patterns. Additionally, we observed the characteristic overshoot in SV after exercise for both breathing patterns (Hughes et al., 1989; Kano et al., 1999; Lässing et al., 2023; Lässing, Hummelmann, et al., 2025).

In conclusion, there is limited comparability to studies on single VMs. Narloch and Brandstater (1995) observed an augmentation of blood pressure across their sequential repetitions. However, no previous studies have monitored SV and cardiac output continuously while performing repetitive BH or VM during exercise.

### 4.2 Pulmonary and metabolic responses to breath‐holding

The ventilatory outcomes exhibited a significant effect of breathing technique in all recorded parameters. The most substantial impact of BH on strength training sessions is a marked reduction in ventilation at all intensities, particularly at the end of sets. This reduction likely reflects both restricted airflow due to glottal closure and the transient mechanical restriction of thoracic expansion during the Valsalva‐like maneuver. This effect is similar to the increased airway resistance caused by using mouthguards during exercise (Lässing et al., 2021). During the recovery period, BH results in significantly higher values of tidal volume, V̇_E_, and V̇O_2_. These post‐exercise overshoots indicate a compensatory response to accumulated carbon dioxide and enhanced chemoreflex drive once normal breathing resumes. Repeated VMs likely reduced V̇_E_ during the sets, as reflected by lower V̇_E_ and V̇O_2_. Given an unchanged overall oxygen demand and lactate production, BH likely shifted the timing, but not the magnitude, of metabolic gas exchange. Previous research indicates that increased cardiopulmonary strain due to higher repetitions of resistance exercise is associated with greater post‐exercise effort (Lässing, Hummelmann, et al., 2025). Nevertheless, Laginestra et al. (2023) revealed that metabolite accumulation sensitizes mechanoreflex‐induced cardiopulmonary responses, independent of biological sex, highlighting a divergent mechanism. Given a constant oxygen demand and lactate production during exercise, reflexively increased post‐exercise V̇_E_ and carbon dioxide output are therefore evident during the rest intervals. This aligns with our observation that BH delays but amplifies ventilatory recovery rather than altering total gas exchange. Jansen et al. (2007) have also reported lower V̇O_2_ values during exercise, with the highest values occurring during the first 40–70 s after a strength training load. In contrast, several authors have reported elevated V̇O_2_ during recovery intervals compared to resting values, but have not observed a visible overshoot (Braun et al., 2005; Lässing et al., 2023; Lässing, Hummelmann, et al., 2025; Pafili et al., 2010; Vilaça‐Alves et al., 2016). Our continuous cardiopulmonary monitoring confirms that these transient overshoots occur systematically after repeated BH, providing new evidence for breathing‐pattern‐dependent modulation of ventilatory dynamics.

To date, no comparable pulmonary data specifically on the use of repetitive short BH or the VM during low‐intensity resistance training in women are available. Under CB, V̇O_2_ values during squats were consistent with previous reports (33%–47% of maximum V̇O_2_) (Collins et al., 1991; Haramura & Takai, 2021; Jansen et al., 2007), classifying the exercise as low‐to‐moderate intensity according to ESC guidelines (Pelliccia et al., 2021), whereas V̇O_2_ during BH remained slightly lower. Similarly, low lactate concentrations corroborate the predominantly aerobic nature of the load (de Sousa et al., 2012; Haramura et al., 2017; Martorelli et al., 2015; Maté‐Muñoz et al., 2015) and were unaffected by BH. However, the significantly higher perceived exertion under BH conditions indicates an altered sensory experience of effort, likely reflecting the transient mismatch between cardiovascular drive and ventilatory output.

In summary, breath‐holding during resistance training, even at low intensity, results in significantly altered breathing patterns, with a lower ventilatory response during repetitions and a compensatory increase in post‐exercise V̇_E_. Together with concurrent elevations in HR and BP, these findings illustrate a coherent physiological mechanism: Mechanical constraints during BH transiently elevate cardiovascular stress and suppress V̇_E_, which, in turn, triggers a reflexive hyperventilatory and circulatory recovery response once normal breathing resumes.

Despite well‐documented sex‐specific physiological differences, our findings indicate that the fundamental hemodynamic and ventilatory responses to BH are present even at low exercise intensities in women. However, the magnitude of these responses in our female participants was notably lower than that observed in men, suggesting that, while the underlying mechanisms are consistent across sexes, the absolute physiological load may be modulated by sex‐specific factors such as vascular compliance, muscle mass, or autonomic regulation. Accordingly, this study highlights the continued need to include female participants in clinical and exercise physiology research.

## 5 STUDY LIMITATIONS

This study has several limitations that should be taken into consideration. The relatively small sample size has limited statistical power, increasing the likelihood of a Type II error, particularly for parameters that were not included in the sample size calculation. However, no data were available on SV or cardiac output related to the breathing techniques used during strength training.

In addition, the sample consisted exclusively of young, healthy females, which limits the generalizability of the findings to clinical populations, such as patients with cardiovascular or pulmonary diseases participating in rehabilitative exercise programs. Studies have shown differences in blood pressure regulation between sexes at rest and during dynamic stress (Bassareo & Crisafulli, 2020; Bauer et al., 2021; Briant et al., 2016; Williams et al., 2017). It remains essential to study a male collective to identify gender‐specific regulatory differences. Also, additional research is needed to investigate these effects in clinical cohorts.

Due to the use of a spirometry mask, it was not possible to accurately control for the BH. As exhalation time was derived solely from the turbine signal and inhalation time was calculated as the interval between consecutive exhalations, post‐inspiratory BH was inherently included in the inhalation time. Exhalation was monitored based on audible cues and recorded respiratory times, which may have introduced variability in respiratory patterns. No intrathoracic pressures were measured, and arterial blood pressure was not continuously monitored. Impedance cardiography may be associated with limited precision and a potential variance of absolute cardiac parameters, particularly during strenuous exercise (Siebenmann et al., 2015). However, validation studies report acceptable agreement with reference methods (Charloux et al., 2000; Hassan‐Tash et al., 2023), supporting its use in physiological studies and its major advantage of non‐invasive measurement during exercise.

The exercise intensity applied was relatively low. Prior research indicates that more pronounced physiological responses may occur at higher intensities, suggesting that some effects might have been underestimated in the present study. Nevertheless, our results from low‐intensity strength training sessions revealed substantial differences among the various breathing techniques. Ventilation measurements may have been biased by the 10‐s averaging interval. However, the results for inspiratory and expiratory time data support the validity of the analysis.

Finally, the limited availability of comparable studies focusing on female participants restricts the ability to contextualize and interpret the results. Future studies should aim to include more diverse populations and a broader range of intensities to strengthen the external validity of the results.

## 6 CONCLUSIONS AND PRACTICAL IMPLICATIONS

This study demonstrates that breath‐holding (BH) during strength training significantly alters cardiovascular and pulmonary responses in young, healthy women, even at low‐to‐moderate exercise intensities. BH led to markedly elevated SBP and rate‐pressure product, indicating increased cardiac workload regardless of load intensity. These effects are likely due to the combined impact of elevated intrathoracic and intra‐abdominal pressures, increased peripheral resistance, and, consequently, a heightened pressor reflex response. While SV was not significantly different between breathing patterns, a pronounced overshoot in SV and cardiac output was observed post‐exercise, suggesting transient hemodynamic suppression during exercise. In contrast to cardiovascular responses, pulmonary responses to BH were associated with reduced ventilatory efficiency and V̇O_2_, without metabolic differences.

These findings suggest that even at lower loads, BH can impose significant cardiovascular strain, with clinical relevance in populations with compromised cardiac function. In healthy young women, the observed responses did not reach critical levels, suggesting that, under controlled, brief application, BH could serve as a safe and feasible breathing technique during strength training. Given the lack of comparable data in women, this study provides important initial insights but also highlights the need for further research.

## AUTHOR CONTRIBUTIONS

**Sonja Hummelmann:** Conceptualization; data curation; formal analysis; investigation; methodology; project administration; validation; visualization. **Maxi Kramer:** Investigation. **Ulrich Laufs:** Data curation; formal analysis; validation; visualization. **Sven Fikenzer:** Data curation; formal analysis; validation; visualization. **Johannes Lässing:** Data curation; formal analysis; supervision; validation; visualization. **Roberto Falz:** Conceptualization; data curation; formal analysis; methodology; supervision; validation; visualization.

## FUNDING INFORMATION

No funds, grants, or other support were received.

## CONFLICT OF INTEREST STATEMENT

The authors have no competing interests to declare and have no relevant financial or non‐financial interests to disclose.

## ETHICS STATEMENT

All procedures described in this study have been performed in accordance with the ethical standards of the responsible committee on human experimentation (institutional and national) and with the principles outlined in the Declaration of Helsinki, as revised in 2013. Written informed consent has been obtained from all participants. The protocol has been approved by the Ethics Committee of the Medical Faculty of the University of Leipzig (023/23‐ek).

## Data Availability

The raw data for the results presented in this manuscript are available from the corresponding author upon reasonable request.
